# Auxin perception in Agave is dependent on the species’ Auxin Response Factors

**DOI:** 10.1038/s41598-020-60865-y

**Published:** 2020-03-02

**Authors:** Víctor J. Cancino-García, Jorge H. Ramírez-Prado, Clelia De-la-Peña

**Affiliations:** Unidad de Biotecnología, Centro de Investigación Científica de Yucatán, Calle 43 # 130 × 32 y 34, Col. Chuburná de Hidalgo, 97205 Mérida, Yucatán México

**Keywords:** Gene ontology, Plant biotechnology, Auxin

## Abstract

Auxins are one of the most important and studied phytohormones in nature. Auxin signaling and perception take place in the cytosol, where the auxin is sensed. Then, in the nucleus, the auxin response factors (ARF) promote the expression of early-response genes. It is well known that not all plants respond to the same amount and type of auxins and that the response can be very different even among plants of the same species, as we present here. Here we investigate the behavior of ARF in response to various auxins in *Agave angustifolia* Haw., *A*. *fourcroydes* Lem. and *A*. *tequilana* Weber var. Azul. By screening the available database of *A*. *tequilana* genes, we have identified 32 *ARF* genes with high sequence identity in the conserved domains, grouped into three main clades. A phylogenetic tree was inferred from alignments of the 32 Agave ARF protein sequences and the evolutionary relationship with other species was analyzed. *AteqARF* 4, 15, 21, and 29 were selected as a representative diverse sample coming from each of the different subclades that comprise the two main clades of the inferred phylogenetic reconstruction. These *ARF*s showed differential species-specific expression patterns in the presence of indole-3-acetic acid (IAA) and 2,4-dichlorophenoxyacetic acid (2,4-D). Interestingly, *A*. *angustifolia* showed different phenotypes in the presence and absence of auxins. In the absence of auxin, *A*. *angustifolia* produces roots, while shoots are developed in the presence of IAA. However, in the presence of 2,4-D, the plant meristem converts into callus. According to our results, it is likely that *AteqARF15* participates in this outcome.

## Introduction

Auxins are a very simple group of plant growth regulators with a very complex set of activities^1^. Auxins appear to be a set of molecules with limitless powers; they give rise to a vast range of specific developmental outputs^2^. The canonical auxin signaling allows auxin to respond in different ways depending on concentration and context^3,4^. The auxin binds to the TIR1/AFB family of auxin receptors, where it works as a cement between the Aux/IAA transcriptional repressor family and the TIR1/AFB receptor^5^. There are different possible Aux/IAA TIR1/AFB combinations that give rise to the wide range of effects produced by auxins^2,3^. Auxins are involved in numerous physiological and developmental processes, such as organogenesis, cellular elongation, apical dominance, gravitropism, root formation, flower morphogenesis, embryo development, and others^6–8^. The auxins indole-3-acetic acid (IAA), indole-3-butyric acid (IBA), phenyl acetic acid (PAA) and 4-chloro-indole-3-acetic acid (4-Cl-IAA)^9–11^ are all auxins previously characterized in plant tissues. However, there are also auxin analogues, such as naphthalene-1-acetic acid (NAA) and 2,4-dichlorophenoxyacetic acid (2,4-D)^1^. At different concentrations, auxins can be used to induce dedifferentiation and redifferentiation of tissues, and also to promote rooting in plants^12–16^. The auxinic herbicides, such as dicamba (3,6-dichloro-2-pyridinecarboxylis acid), dichloroprop [(±)-2-(2,4-dichlorophenoxy) propionic acid], 2,4-D [(2,4-dichlorophenoxy) acetic acid], and several more are described as synthetic auxins or growth regulators with herbicidal action or herbicides with growth regulator activity^11,17,18^. It has been reported that the effect of 2,4-D is 300 times more potent than IAA on the growth of plants^11^. The major characteristic shared by auxins and auxinic herbicides is the fact that the positive charge of the indole nitrogen is separated by 5.5 Å from a negatively charged substituent (the carboxyl group) on a flat lipophilic group/ring system^11^.

The genes that encode the enzymes and proteins needed for synthesis, transport, and signaling of auxins have been identified mainly in maize, rice and *Arabidopsis thaliana*^19–22^. Some of the early genes transcribed due to auxin response are *Auxin/IAA* (*Aux/IAA*), *Gretchen Hagen* 3 (*GH3*) and *SMALL AUXIN UP RNA* (*SAUR*), which regulate the physiology of the plant by modulating the interaction of the auxin-responsive elements (AuxRes) in some genes^23–25^. The Aux/IAA proteins contain four conserved domains (I-IV). The role of domain I has been reported as a potential active repression domain in Arabidopsis^26^. Domain II is required for Aux/IAA degradation, which is essential for normal auxin signaling^27,28^. Domains III and IV work like a domain of protein-protein interaction allowing the formation of homo- and heterodimers among Aux/IAA and repressing the activity of the Auxin Response Factors (ARFs)^26,29^.

ARFs are transcription factors that bind to the AuxRes, regulating the transcription of downstream genes^25,30^. The protein structures of the 23 ARFs reported in Arabidopsis are divided into three main domains. One is an N-terminal B3-type DNA binding domain (DBD), which binds to the AuxRes, followed by a domain rich in glutamine-serine-leucine (QSL) or serine-proline-leucine/glycine (SPLG) that functions as an activation or repression domain, respectively. The third domain is the carboxy-terminal dimerization domain, which is homologue to the domains III and IV in the Aux/IAA proteins to form heterodimers^31^. Of the 23 ARFs, five (ARF5–8 and 19) have activation domains and, therefore, can activate the transcription of auxin response genes, while the rest of the ARFs (18) function as transcriptional repressors^31–33^.

ARFs have important functions in lateral root growth^34,35^, embryogenesis^36,37^, leaf expansion^38,39^ and fruit development^40,41^, and they have also been studied in response to different auxin concentrations^42^. In an Arabidopsis transcriptomic study, Paponov *et al*.^42^ found that in response to different concentrations of auxins, from very low (0.1 μM) to very high (10 μM), *ARFs* 4, 16 and 19 were up-regulated in response to auxin. Of these three, only *ARF19* was sensitive to a very low auxin concentration. ARF19 is considered to be a transcriptional activator due to its QSL-rich characteristic domain^31^ and it has been also considered to be a tissue-specific amplifier of the auxin signal^42^. The study of different *ARFs* in plant tissue culture has been justified by the use of exogenous auxins in order to develop either organogenesis or embryogenesis in important plants^37,43–46^. However, ARFs in non-model plants or from different species from the same genus have been poorly investigated.

*Agaves* are of great importance for their role as a source of raw material in the fiber industry, such as *A*. *fourcroydes* (pentaploid). In the liquor industry, e.g., the production of tequila^47^, *A*. *tequilana* Weber (Var. Azul, diploid) has been used with great success, and additionally is designated as a plant that originated in Mexico (NOM-V-1978). *A*. *angustifolia* Haw. (Var. Bacanora, pentaploid) has been used for mezcal production^48^. Although the genome of *Agaves* has not been sequenced due to its large size and complexity^49,50^, there are reports describing transcriptome analysis in *A*. *tequilana*^49,51^, *A*. *deserti*^51^ and *A*. *sisalana*^52^. The aim of this work is to determine how different auxins affect the growth and phenotype of different *Agave* species (*A*. *angustifolia*, *A*. *fourcroydes* and *A*. *tequilana*) and to elucidate the effects on short- and long-term cultivation of IAA and 2,4-D targeted toward two ARF with domains related to repression, *AteqARF4* and 21, and two ARF related to the activation of expression, *AteqARF15* and 29.

## Results

### Identification of ARF in *Agave spp*. revealed by gene structure and protein analysis

**Figure 1:**
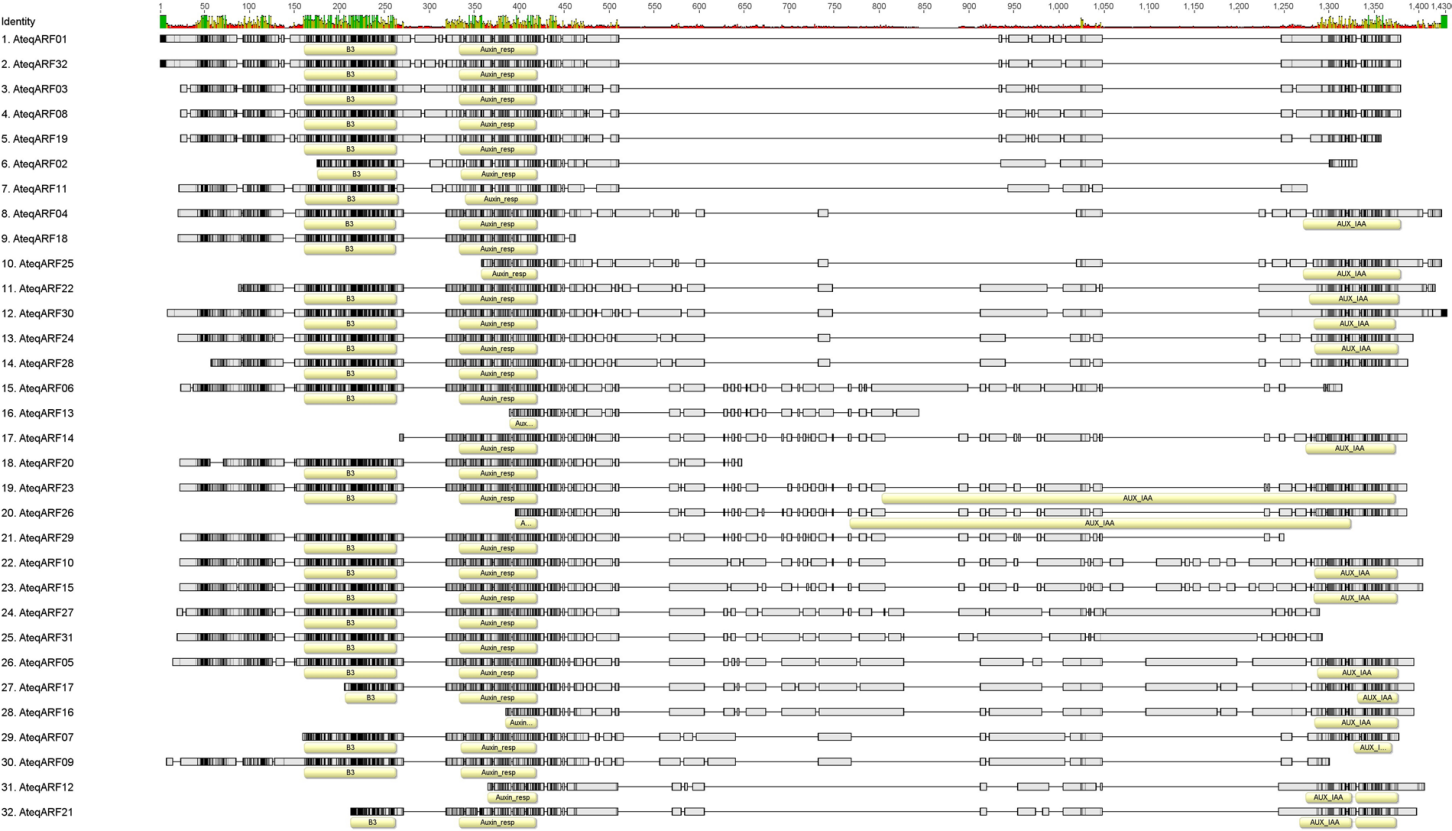
Graphical representation of the Multiple Sequence Alignment (MSA) of the 32 putative ARF proteins from *A*. *tequilana*. Yellow boxes: Conserved domains predicted by Interproscan against Pfam signatures database; Gray to black boxes: blocks of conserved amino acids (darker = more conserved); thin horizontal lines: alignment gaps. The top histogram indicates percentage of identity at each position.

Identification of the ARFs present in *Agave* was done from an *Agave* transcriptome database^51^ by homology to known ARFs. Using as probes the 23 well-characterized members of the *A*. *thaliana* ARF family, we found 32 putative Agave ARF transcripts (Table 1). These sequences were aligned to find the conserved domains (Fig. 1). Three characteristic ARF domains were found: the DNA binding domain (DBD; B3), the auxin response domain, and the AUX-IAA domain. According to Zhang *et al*.^53^, sequences that do not have the DBD domain are considered to be pseudogenes and cannot be functional. Therefore, we scanned the proteins encoded by the transcripts to annotate the presence of conserved domains and discarded those that did not contain a DBD domain. Of the 32 putative ARF sequences, only 23 have the complete DBD domain, three have an incomplete DBD sequence and six do not have a DBD domain. ARFs with incomplete or missing domains could be pseudogenes or be due to technical problems of the transcriptome assembly.

**Table 1:** *ARF* gene family in Agave.

AteqARF	Locus name	Accession number	Cds length (bp)	Amino acids (aa)	Molecular weight (kda)	Isoelectric point
1	Locus1985413v1rpkm13.02	GAHU01042083.1	2706	1,283	75.849	7.19
2	Locus31465v1rpkm5.34	GAHU01064529.1	1622	1,060	37.274	5.59
3	Locus36268v1rpkm3.65	GAHU01073181.1	3207	1,281	75.926	7.03
**4**	**Locus8030v1rpkm37.42**	**GAHU01017508.1**	**2573**	**1,324**	**78.413**	**6.19**
5	Locus35770v1rpkm3.80	GAHU01072281.1	4051	1,304	111.996	4.95
6	Locus9114v1rpkm33.06	GAHU01019718.1	3072	1,180	90.324	7.39
7	Locus17266v1rpkm15.86	GAHU01036963.1	2318	1,143	72.329	8.26
8	Locus28611v1rpkm6.66	GAHU01059165.1	3379	1,281	75.812	6.82
9	Locus12504v1rpkm23.71	GAHU01027178.1	2674	1,204	78.265	6.89
10	Locus29753v1rpkm6.09	GAHU01061328.1	3672	1,306	109.485	6.65
11	Locus21203v1rpkm11.76	GAHU01044804.1	2091	1,288	58.477	6.44
12	Locus7292v1rpkm41.07	GAHU01016017.1	1833	956	45.054	5.42
13	Locus7068v1rpkm42.25	GAHU01015477.1	833	387	33.123	7.51
14	Locus4331v1rpkm66.11	GAHU01009791.1	2357	1,038	63.631	4.95
**15**	**Locus26307v1rpkm7.95**	**GAHU01054801.1**	**3638**	**1,306**	**107.541**	**6.44**
16	Locus54454v1rpkm1.35	GAHU01101468.1	2524	919	77.804	4.37
17	Locus38447v1rpkm3.09	GAHU01076973.1	3011	989	84.153	4.47
18	Locus8706v1rpkm34.58	GAHU01018880.1	1771	466	41.919	7.27
19	Locus24217v1rpkm9.35	GAHU01050701.1	2774	1,246	71.251	6.94
20	Locus15910v1rpkm17.63	GAHU01034284.1	1650	572	49.658	7.52
**21**	**Locus9914v1rpkm30.43**	**GAHU01021381.1**	**2349**	**1,087**	**53.397**	**6.21**
22	Locus4828v1rpkm60.36	GAHU01010818.1	3384	1,188	70.511	8.39
23	Locus14749v1rpkm19.44	GAHU01031919.1	3017	1,288	89.347	6.16
24	Locus31272v1rpkm5.41	GAHU01064202.1	2391	1,297	80.530	6.85
25	Locus4067v1rpkm69.76	GAHU01009186.1	2023	963	45.409	5.10
26	Locus23562v1rpkm9.86	GAHU01049445.1	2054	911	55.154	4.67
27	Locus8507v1rpkm35.35	GAHU01018502.1	3142	1,327	110.038	7.18
28	Locus44034v1rpkm2.15	GAHU01086204.1	2252	1,239	73.687	7.43
**29**	**Locus6215v1rpkm47.55**	**GAHU01013606.1**	**2478**	**1,355**	**74.284**	**6.53**
30	Locus15254v1rpkm18.61	GAHU01033026.1	2896	1,347	94.016	7.27
31	Locus8241v1rpkm36.50	GAHU01017933.1	3414	1,331	111.660	7.54
32	Locus24796v1rpkm8.95	GAHU01051896.1	2869	1,304	78.523	7.29

### Phylogenetic analysis reveals ARF homology with other plants

**Figure 2:**
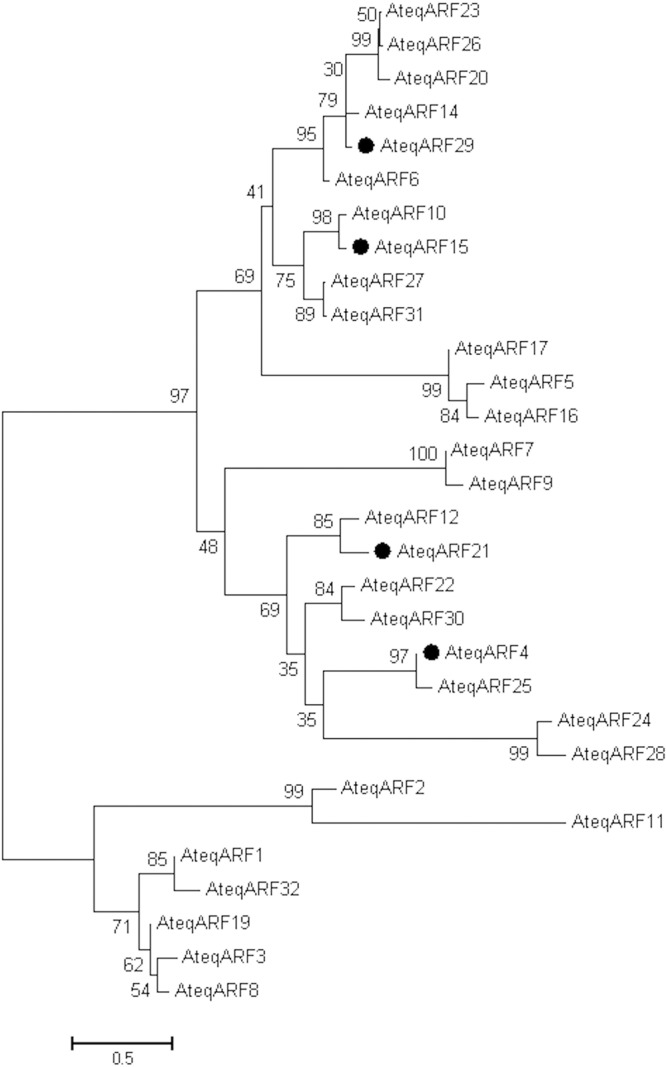
Phylogenetic analysis of the ARF of *Agave tequilana*. Phylogeny was calculated from a MSA of the 30 putative *A*. *tequilana* ARFs (two highly truncated ARFs were removed). The evolutionary history was inferred using the Minimum Evolution method^81^. The optimal tree with the sum of branch length = 11.98949640 is shown. The percentage of replicate trees in which the associated taxa clustered together in the bootstrap test (1,000 replicates) are shown next to the branches^82^. The tree is drawn to scale, with branch lengths in the same units as those of the evolutionary distances used to infer the phylogenetic tree. The evolutionary distances were computed using the JTT matrix-based method^80^ and are in the units of the number of amino acid substitutions per site. The rate variation among sites was modeled with a gamma distribution (shape parameter = 1.219). The ME tree was searched using the Close-Neighbor-Interchange (CNI) algorithm^83^ at a search level of 2. The Neighbor-joining algorithm^84^ was used to generate the initial tree. The analysis involved 30 amino acid sequences. All positions containing gaps and missing data were eliminated. There were a total of 74 positions in the final dataset. Evolutionary analyses were conducted in MEGA7^85^. ARFs selected for gene expression analyses are marked by solid black circles.

**Figure 3:**
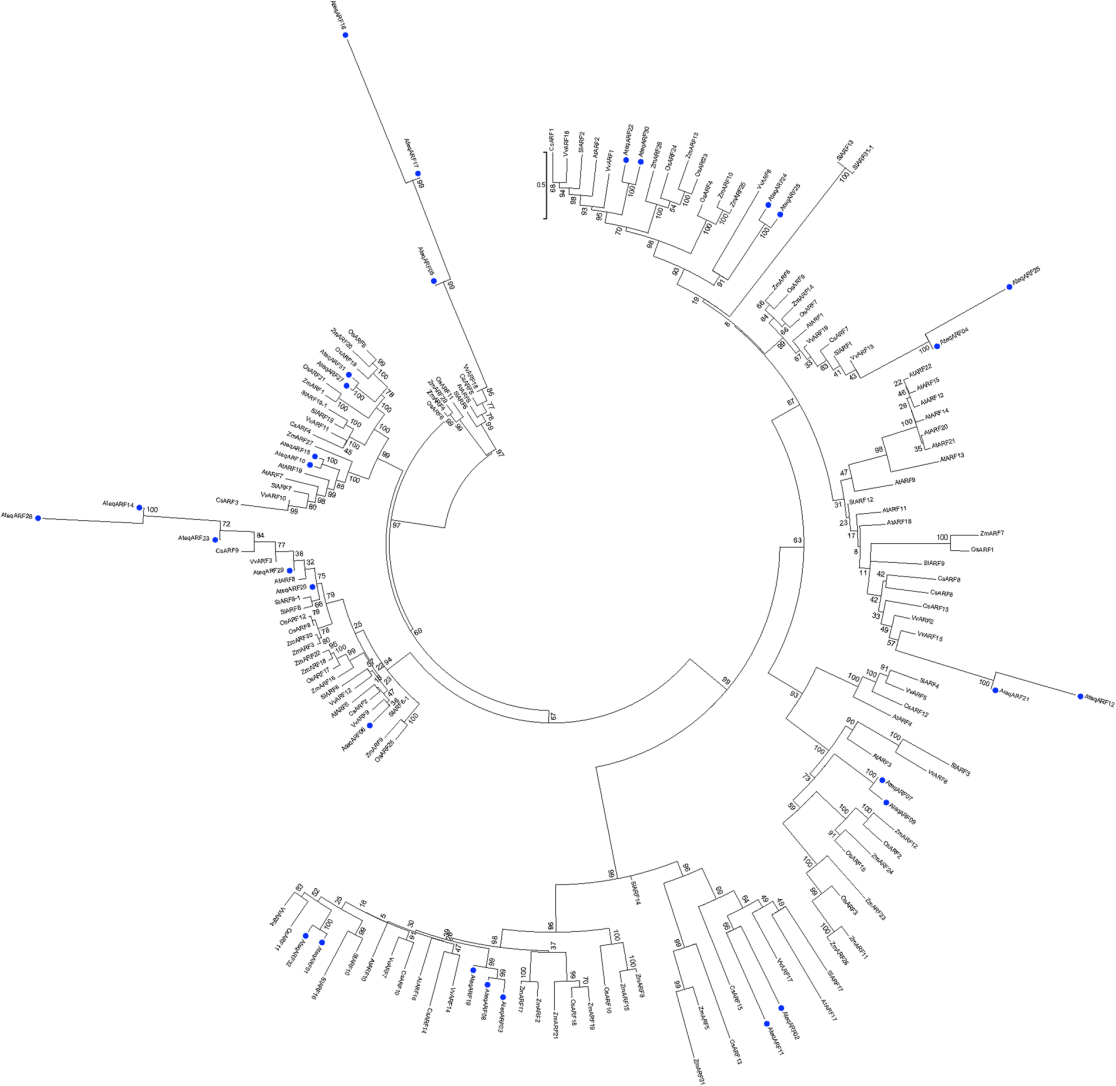
Phylogenetic relationship of the ARF of Agave to other species. Phylogeny was calculated from an MSA of the 30 putative *A*. *tequilana* ARFs (marked by solid blue circles) plus 127 ARFs from seven previously reported plant species (15 *ARF* of *Cucumis sativus*^54^, 19 of *Vitis vinifera*^55^, 20 of *Solanum lycopersicum*^56^, 22 of *A*. *thaliana*^57^, 20 in *Oryza sativa*^58^, and 31 in *Zea mays*^59^). The evolutionary history was inferred using the Minimum Evolution method^81^. The optimal tree with the sum of branch length = 52.16578416 is shown. The tree is drawn to scale, with branch lengths in the same units as those of the evolutionary distances used to infer the phylogenetic tree. The evolutionary distances were computed using the JTT matrix-based method^80^ and are in the units of the number of amino acid substitutions per site. The rate variation among sites was modeled with a gamma distribution (shape parameter = 1.011). The ME tree was searched using the Close-Neighbor-Interchange (CNI) algorithm^83^ at a search level of 2. The Neighbor-joining algorithm^84^ was used to generate the initial tree. The analysis involved 158 amino acid sequences. All ambiguous positions were removed for each sequence pair. There were a total of 1827 positions in the final dataset. Evolutionary analyses were conducted in MEGA7^85^.

A phylogenetic analysis divided 30 of 32 transcripts into three major clades (Fig. 2). AteqARF18 and AteqARF13 were not used in the analysis due to their truncated structure (Fig. 1). The phylogenetic analysis of the *ARF* of *Agave* was performed by comparing these to the *ARF* from seven others already reported from different plant species, including 15 *ARF* of *Cucumis sativus*^54^, 19 in *Vitis vinifera*^55^, 20 in *Solanum lycopersicum*^56^, 22 in *A*. *thaliana*^57^, 20 in *Oryza sativa*^58^, and 31 in *Zea mays*^59^ (Fig. 3). Interestingly, 13 of the *Agave ARF* genes were grouped with the *ARF* of *V*. *vinifera* (*Vv*). Specifically, *AteqARF12* and *AteqARF*21 were mainly grouped with *VvARF15*; *AteqARF4* and *AteqARF25* were grouped in the same clade with *VvARF13; AteqARF24* and *AteqARF28* were grouped with *VvARF6; AteqARF22* and *AteqARF30* were grouped with *VvARF1; AteqARF6* was grouped with *VvARF9; AteqARF*29 was grouped with *VvARF3;* and *AteqARF5*, *AteqARF16*, and *AteqARF17* were grouped with *VvARF18* (Fig. 3).

### Auxins display phenotypic differences in *Agave* spp

**Figure 4:**
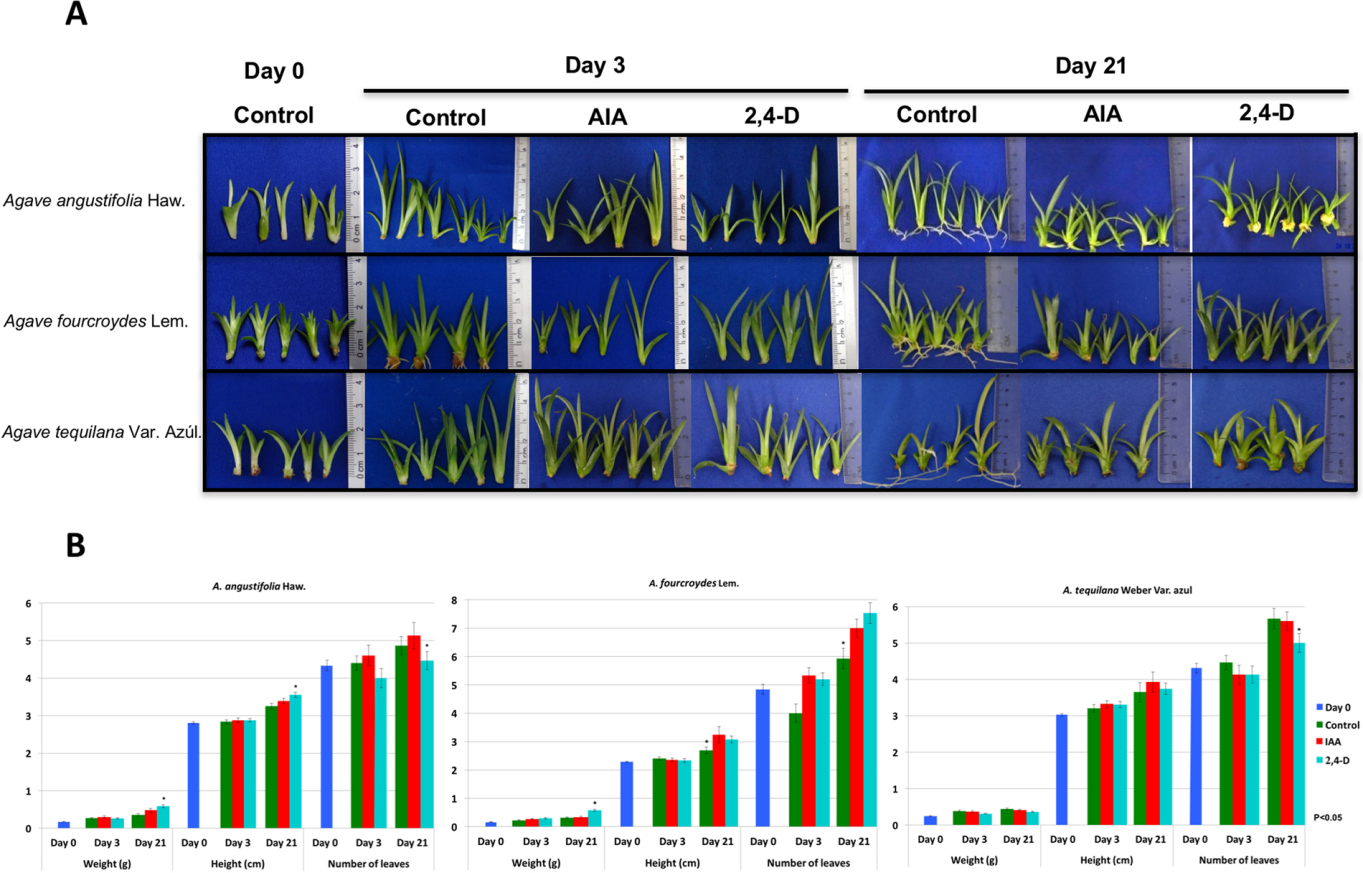
Effect of IAA and 2,4-D on different Agave species. (**A**) Plantlets of *A*. *angustifolia*, *A*. *fourcroydes* and *A*. *tequilana* in the presence and absence (control) of auxins at 0, 3 and 21 days. The plantlets were treated with 0.5 μM of IAA and 2,4-D. (**B**) Phenotypic variation of weight, height and number of leaves in *A*. *angustifolia*, *A*. *fourcroydes* and *A*. *tequilana* in the presence and absence (control) of auxins at 0, 3 and 21 days.

In order to know how different auxins affect the growth and phenotype of different *Agave* species (*A*. *angustifolia*, *A*. *fourcroydes* and *A*. *tequilana*), all plants were exposed to the same conditions during days 3 and 21 (see Materials and Methods). These concentrations and time points were selected based on previous experiments done in our group^60,61^. All *Agave* plantlets at the beginning of the experiment (control, day 0), were selected to have the same height, on average 2.5 cm (Fig. 4A). After being in contact with 0.5 μM of IAA and 2,4-D for days 3 and 21, the weight, height and number of leaves were recorded (Fig. 4B). Due to its robust nature, plantlets of *A*. *fourcroydes* have more leaves than *A*. *angustifolia* or *A*. *tequilana*. After 3 days in contact with IAA and 2,4-D, *Agave* species started to show important differences in comparison with the control (without auxins). For instance, while *A*. *fourcroydes* started to develop roots in the absence of auxins, in the presence of either IAA or 2,4-D there was no root development, although these plants were thinner than the control (Fig. 4A). On the other hand, *A*. *angustifolia* showed substantial growth variation after 3 days without auxins. It was found that while some *A*. *angustifolia* plantlets did not grow, others grew taller, reaching more than 5 cm in height (Fig. 4B). In the presence of 2,4-D, *A*. *angustifolia* and *A*. *fourcroydes* plantlets grew better and had a greener phenotype than in the presence of IAA. The opposite result happened in *A*. *tequilana*, where the plants grew better in the presence of IAA than in 2,4-D. The most interesting results were observed at 21 days in the presence and absence of the auxins. For instance, in the absence of auxins, *A*. *angustifolia* developed roots, in the presence of IAA the plantlets developed shoots, and in the presence of 2,4-D, the plantlets developed callus (Fig. 4A). It is very likely that the formation of callus contributed to this condition, having more weight (0.586 g) than in the presence of IAA (0.479 g) or the control (0.351 g) (Fig. 4B). These phenotype characteristics did not appear in *A*. *fourcroydes* or *A*. *tequilana*, where roots only developed in the absence of auxins. Although the roots in the control of *A*. *fourcroydes* emerged at day 3, by day 21 *A*. *tequilana* presented the longest roots. It is worth noting that *A*. *tequilana* in the presence of 2,4-D developed plants with wide leaves.

### Gene expression of ARF revealed species-specific sensibility to auxins

**Figure 5:**
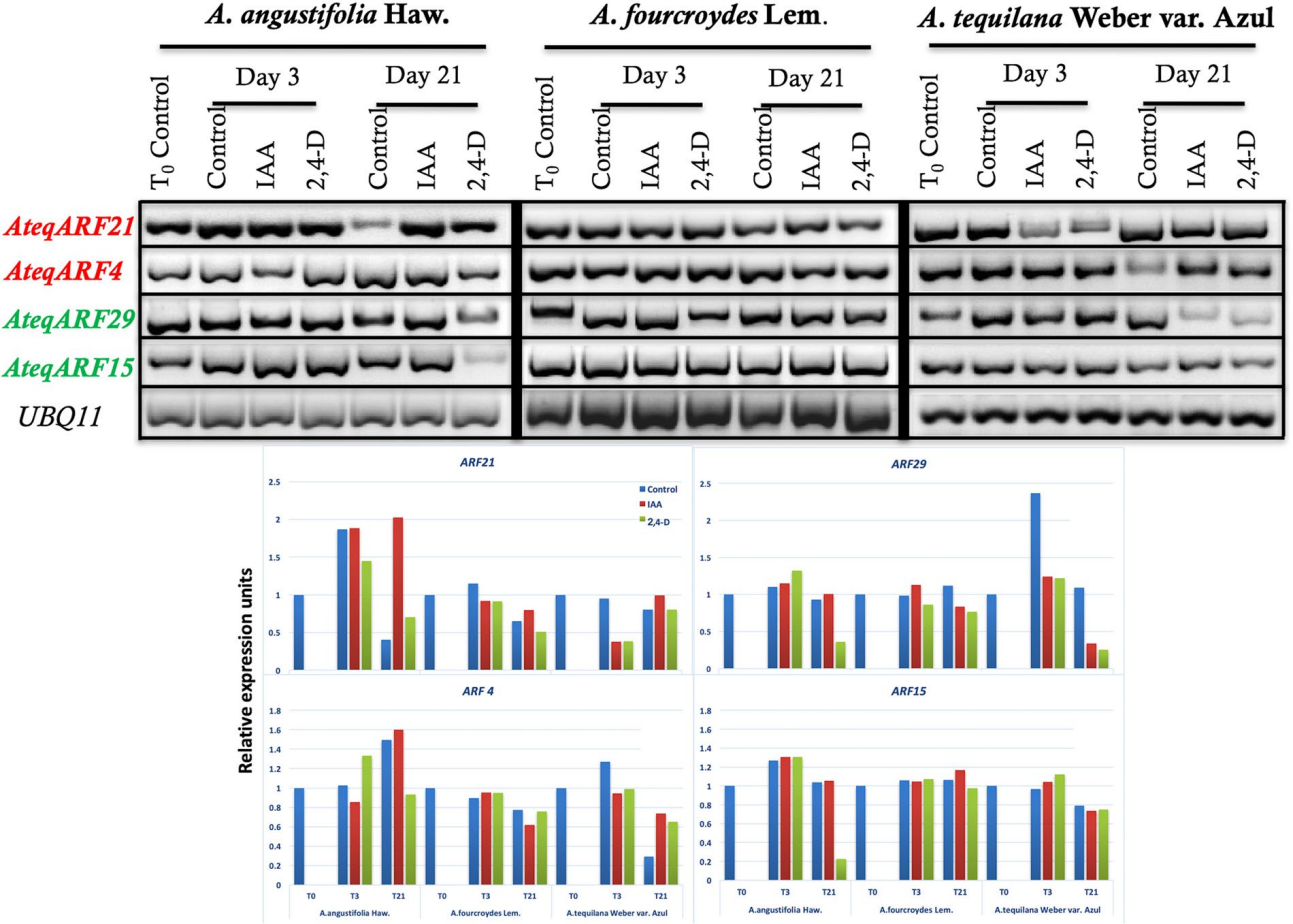
Expression profiles of *ARF* in *Agave angustifolia*, *A*. *fourcroydes* and *A*. *tequilana*. (**A)** Expression of *AteqARF4*, *15*, 21 and 29 as shown by RT-PCR analysis of total RNA samples isolated from plantlets treated with 0.5 μM of IAA and 2,4-D at days 0, 3 and 21. *UBQ11* was used as a reference gene. (**B**) Densitometric analysis of the gene expression shown in A. Relative expression of *AteqARF4*, 15, 21 and 29 were normalized to the constitutive gene *UBQ11*.

To determine the possible effects over time of IAA and 2,4-D on gene expression, we analyzed the expression pattern of two ARF with domains related to repression, *AteqARF4* and *21*, and two ARF related to activation of expression, *AteqARF15* and *29*, after 3 and 21 days in the absence and presence of auxins (Fig. 5). These *ARFs* were selected as a representative diverse sample coming from each of the different subclades that comprise the two main clades inferred from the phylogenetic reconstruction (Fig. 2).

Unexpectedly, *A*. *fourcroydes* did not present important expression changes in the absence or presence of IAA or 2,4-D. Interestingly, in the case of *A*. *angustifolia* and *A*. *tequilana*, the presence of IAA or 2,4-D provoked differential expression for different *ARF*s. For instance, *AteqARF21* had very low expression in *A*. *angustifolia* without auxins at day 21, while in *A*. *tequilana* low expression was detected in the presence of both IAA and 2,4-D at day 3. *AteqARF4* showed low expression only in the absence of auxins in *A*. *tequilana* at day 21. In the case of *AteqARF29*, the transcript levels were very low in 2,4-D at day 21 in *A*. *angustifolia* and in the presence of both IAA and 2,4-D in *A*. *tequilana*. On the other hand, *AteqARF15* only showed low expression in the presence of 2,4-D in *A*. *angustifolia* at day 21 (Fig. 5). Therefore, ARF15 is affected by 2,4-D but only in *A*. *angustifolia*. This opens a new avenue to study auxin regulation in a species-specific manner that will help to develop new strategies to understand plant development.

## Discussion

The family of the *ARF* genes is large and its members have functional redundancy, which creates additional challenges when trying to understand ARF function. Finet *et al*.^62^ reported a complete phylogenetic analysis covering 21 species, including *A*. *thaliana*, *Carica papaya*, *Glycine max*, *Medicago truncatula*, *O*. *sativa*, *V*. *vinifera*, *Z*. *mays* and others in order to find the similitude between and among the ARF of different species. In that analysis, it was found that all ARFs were grouped in three clades. We also found that the 23 ARFs in *Agave* can be grouped in these three major clades (Fig. 2). With a phylogenetic analysis, we found that *AteqARF15* was in the same clade as *ARF27* of *Z*. *mays*, *ARF7* of *Solanum lycopersicum*, *ARF7* and 19 of *A*. *thaliana*, *ARF10 of V*. *vinifera and ARF3 of C*. *sativus* (Fig. 3). This similitude seems to be the result of duplication events among the genes and a domain rearrangement due to alternative splicing^63,64^, which has modified the number of *ARF* genes in different species^53,62^. An expression analysis *in silico* of *ZmARF27* showed that this gene in maize is mainly expressed in the seed, tassel and ear^59^, tissues affected by auxin concentration^65–67^. For instance, Chen *et al*.^65^ found that there is a positive correlation between IAA concentration and ear height. On the other hand, Erkoyuncu *et al*.^67^ reported that high concentrations of 2,4-D increase callus formation in maize embryos. We found that 2,4-D was associated with callus formation in *A*. *angustifolia* after 21 days, the day at which *AteqARF*15 was downregulated (Fig. 5). A similar conclusion was made in a study related to callus formation in Arabidopsis and rice, in which it was found that *AtIAA14-ARF7/19* in Arabidopsis is not required for callus initiation but in rice a homolog gene is strictly required^68^.

The number of *ARF* genes in *Agave* (Table 1) is consistent with the number of ARF found in other monocotyledonous species^62^. Studies in Arabidopsis have revealed that ARFs show gene redundancy^69^. Therefore, we selected four representative *ARF*, two repressor-related and two activator-related, to investigate whether different species exposed to auxin could interfere with *ARF* gene expression (Fig. 5). Here, we show that plants from the same genus but different species not only can be susceptible to a different degree in the presence or absence of auxins but also depending on the auxin (IAA or 2,4-D). In all cases, the effect on the expression of the *ARFs* would be different (Fig. 5).

*In vitro* propagation of Agave has solved many of the problems that conventional *Agave* farming has faced, such as a long maturation period and lack of high-quality materials to work with and genetic improvement programs^70–72^. *In vitro* culture represents an excellent alternative for producing a massive number of plants with superior market qualities in a short period of time. However, plant growth regulators have been a challenge to work with in order to determine the best doses for plant growth and propagation, and auxin concentrations have been particularly challenging. The use of concentrations of 2,4-D between 0.05 and 0.5 μM have been related to phenotypic variation in commercial cultivars such as strawberry^73^, soybean^74^ and cotton^75^. In the case of Agave, the concentration of 0.5 μM of 2,4-D provoked the formation of callus in *A*. *angustifolia* while in *A*. *tequilana* and *A*. *fourcroydes* it promoted robustness of the leaves, a finding that indicates different auxin regulation functions in different species. In *A*. *thaliana*, it has been reported that *ARF19* increases its expression as a function of the concentration – the greater the concentration, the higher the expression – and the time in contact with external IAA^42^.

Given that only *A*. *angustifolia* showed callus formation in response to the same auxin stimulus experienced by *A*. *tequilana* and *A*. *fourcroydes*, it is clear that a different mechanism of callus initiation must exist between species. It is important to answer how the mechanism for auxin response differs in one species versus another species and how ARFs can be regulated in order to achieve high-quality plants resistant to developing challenges.

## Materials and Methods

### Plant material and culture conditions

Three different *in vitro*-propagated *Agave* species (*Agave angustifolia* Haw., *A*. *fourcroydes* Lem., and *A*. *tequilana* Weber var. Azul) were used to perform all the analyses. All *Agave* plantlets were cultured in multiplication media supplemented with 2.22 μM BA and 0.1 μM 2,4-D^76^ under photoperiod (12/12 hr) conditions. After six weeks, plantlets 1.5–2 cm in height were selected in order to have a homogenous group of plants. Once plants of the same size from each species were selected, these were kept for eight weeks in Magenta culture boxes filled with 50 mL of Murashige and Skoog media with reduced nitrogen, solidified with 1.75 g/L of Gelrite and without growth regulators in order to avoid contact with exogenous growth regulators before the treatments. After a two-month period without auxins, each *Agave* species was cultured with either of two different types of auxins (IAA and 2,4-D) at 0.5 μM and without auxins (Control). In each condition for all *Agave* species the weight, height and number of leaves were evaluated at days 0, 3 and 21. These concentrations and time points were selected based on previous experiments done in our group^60,61^.

### Bioinformatic and phylogenetic analysis

Putative ARF transcripts in the reported transcriptome of *A*. *tequilana* at NCBI’s database^51^ were identified by means of a homology search using the BLASTN algorithm and the 23 *A*. *thaliana ARF*s as queries. The theoretical coding sequences and proteins for the transcripts were predicted with FGENESH+^77^, using the closest *A*. *thaliana* ARF protein sequence as a homologous reference for each putative transcript. A multiple sequence alignment (MSA) of the putative 30 *A*. *tequilana* ARF proteins, plus the previously reported ARFs from seven other plant species [15 *ARF* of *Cucumis sativus*^54^, 19 of *Vitis vinifera*^55^, 20 of *Solanum lycopersicum*^56^, 22 of *A*. *thaliana*^57^, 20 in *Oryza sativa*^58^, and 31 in *Zea mays*^59^], was then obtained using the MAFFT algorithm^78^ implemented on the software Geneiuos® R7 (http://www.geneious.com) with option E-INS-I^79^. A phylogeny of the *A*. *tequilana* and *A*. *thaliana* ARF proteins was inferred by the Minimum Evolution method using the JTT matrix-based model^80^ with a gamma distribution (shape parameter α = 1.011), and 1,000 bootstrap repetitions as a test of confidence level. The best amino acid substitution model was found using the best-fit test implemented in MEGA7. There were 1,827 amino acid positions on the dataset. Ambiguous data were removed for each sequence pair. For structural analysis and determination of conserved domains, the aligned sequences were tested against the databases of Pfam (http://pfam.xfam.org/), Gene3D (http://gene3d.biochem.ucl.ac.uk/Gene3D/), Interpro (http://www.ebi.ac.uk/interpro/), SMART (http://smart.embl-heidelberg.de/), PANTHER (http://www.pantherdb.org/), and Superfamily (http://supfam.org/SUPERFAMILY/hmm.html), using the InterProScan plugin for Geneious® R7.

Highly truncated *A*. *tequilana* ARF proteins missing entire domains due to incomplete transcripts were removed from the original multiple alignment as well as all non-A. *tequilana* ARF proteins. A second phylogeny was inferred from the resulting dataset as above with the following changes: gamma distribution α = 1.219 and complete deletion of ambiguous or missing data (gaps). There was a total of 30 amino acid sequences with 74 positions in the final dataset.

### Primer design

Primers for the different ARF genes of the three selected *Agave* species were designed based on the identified sequences in the *A*. *tequilana* transcriptome^51^. Primer sequences for RT-PCR are listed in Table S1.

### RT-PCR analysis

Total RNA was extracted from 0.3 g of leaf tissue from each plant (*A*. *angustifolia*, *A*. *fourcroydes*, and *A*. *tequilana*) and every condition (control, IAA, and 2,4-D) at day 0, 3, and 21 using the BRL Trizol reagent (Invitrogen) and re-purified with the Qiagen RNeasy Mini Kit, following the manufacturer’s instructions. RT reactions were performed in a 20 µl volume containing 5 µg of total RNA and 200 units of the M-MLV Reverse Transcriptase (Invitrogen), following the manufacturer’s conditions. Platinum Taq polymerase (Invitrogen) was used for the PCR reaction, and conditions were as follow: 5 min at 95 °C, followed by 35 cycles of 95 °C for 40 s, 62 °C for 40 s, and 72 °C for 2 min, and a final cycle of 72 °C for 10 min. Ubiquitin (*UBQ11*) was used as a constitutive gene control. The primer sequences of the *ARF* genes are listed in Table S1. The PCR products were electrophoresed in a 1.5% agarose gel and the identity was confirmed through sequencing (Clemson University Genomic Institute). Each RT-PCR was conducted twice with three biological replicates.

The densitometric analyses for band intensities were performed using ImageQuant™ software. Intensities of *ARF*s in each *Agave* species were normalized versus the *UBQ11*. The fold induction of each condition was calculated relative to the T_0_ control sample.

### Statistical analysis

All data were analyzed using the Origin 8 program. The analysis of variance (ANOVA) was used to calculate the statistical significance and means ± SE. Significant differences were determined using the Tukey’s test at *P* < 0.05.

## Supplementary information

Table S1.
