# Supplementary material for: Auxin perception in Agave is dependent on the species’ Auxin Response Factors
Source: Sci Rep. 2020 Mar 2;10:3860. doi: 10.1038/s41598-020-60865-y (PMC7052169; doi:10.1038/s41598-020-60865-y)
Supplement: Supplementary file 1 — Table S1. [file 41598_2020_60865_MOESM1_ESM.docx]

***Auxin perception in Agave is dependent on the species’ Auxin Response Factors***

**Víctor J. Cancino-García, Jorge H. Ramirez-Prado and Clelia De-la-Peña^&^**

Unidad de Biotecnología, Centro de Investigación Científica de Yucatán, Calle 43 # 130 x 32 y 34, Col. Chuburná de Hidalgo, 97205 Mérida, Yucatán, México

^&^To whom reprint requests should be addressed: Unidad de Biotecnología, Centro de Investigación Científica de Yucatán, Col. Chuburná de Hidalgo, 97205 Mérida, Yucatán, México. Phone (999)942-8330; Fax: (999)981-3900; Email: [clelia@cicy.mx](mailto:clelia@cicy.mx)

Table S1. Primers used for RT-PCR analysis of *ARF* genes.

| *Gene* | Sequence (5´ 3’) | Size of product (bp) | Tm °C |
| --- | --- | --- | --- |
| *AteqARF21* | F: ACCAAAGGAGATCCAGAGCAAGCA  R: GCAGCAGCATTATCACAACCACCA | 372 | 58 |
| *AteqARF15* | F: AGTTGCGGCGTGCTGTTAGT  R: TCGGTGGAGAGGCAGATGTTGA | 459 | 58 |
| *AteqARF4* | F: CTATGGTGAAGCTGATGACCAG  R: AGCCTGCGGTAAGGGTTTCACA | 603 | 58 |
| *AteqARF29* | F: TTCCGAGGTCAACCCAAAAGGCAT  R: TGCTCATTTCCAACCGCCAAAGGT | 755 | 59 |
